# Cold-stored platelets: revisiting assumptions and addressing variability to support implementation

**DOI:** 10.3389/fmed.2025.1743750

**Published:** 2025-12-15

**Authors:** Janhavi Mahajan, Matthew P. Padula, Denese C. Marks, Lacey Johnson

**Affiliations:** 1 Research and Development, Australian Red Cross Lifeblood, Alexandria, NSW, Australia; 2 School of Life Sciences, University of Technology Sydney, Ultimo, NSW, Australia; 3 Sydney Medical School, University of Sydney, Camperdown, NSW, Australia; 4 School of Science, RMIT University, Melbourne, VIC, Australia

**Keywords:** platelet, cold-stored, transfusion, refrigeration, storage

## Abstract

Platelets are transfused to patients to prevent and stop bleeding. Conventionally, platelets are stored at room temperature (RT; 20–24 °C), however, this limits their shelf life to 5–7 days, due to an increased risk of bacterial proliferation at RT. In recent years, cold storage (2–6 °C) of platelets has regained interest, largely due to the potential to extend the shelf life up to 21 days. The historical use of cold-stored platelets and decades of foundational research has made their (re)implementation possible, with cold-stored platelets already being transfused in several countries, including the United States and Norway. However, as efforts continue to expand implementation, it is becoming increasingly evident that variations in processing methods from collection to transfusion, including the collection platform, storage solution and additional component modifications, may alter platelet characteristics during cold storage. This variability is largely overlooked and there is a need to recognize how these differences may affect clinical outcomes post-transfusion. This review outlines the assumptions that have been made regarding cold-stored platelets and discusses areas that require further consideration in an effort to inform future research and best practice.

## 1 Introduction

Platelets for transfusion are stored at room temperature (RT; 20–24 °C) with continuous agitation to facilitate gas exchange (1). These storage conditions have been standard practice since the 1970s, when it was identified that RT storage improves *in vivo* circulation time (8–10 days) in comparison to cold storage (2–4 days), which was the method used for platelet preservation prior to this time (2–4). Accordingly, for more than 50 years, platelets have been exclusively stored at RT, as it provides the best option for prophylaxis – the most common clinical indication for receiving a platelet transfusion (5). However, the trade-offs with RT storage include an increased risk of bacterial growth and a gradual decline in quality parameters as storage progresses, which limits the shelf life of platelet components to 5–7 days (6), depending on institutional regulations. This short shelf life contributes to platelet outdating and product wastage, making inventory management challenging, especially in rural and remote areas.

**Figure 1:**
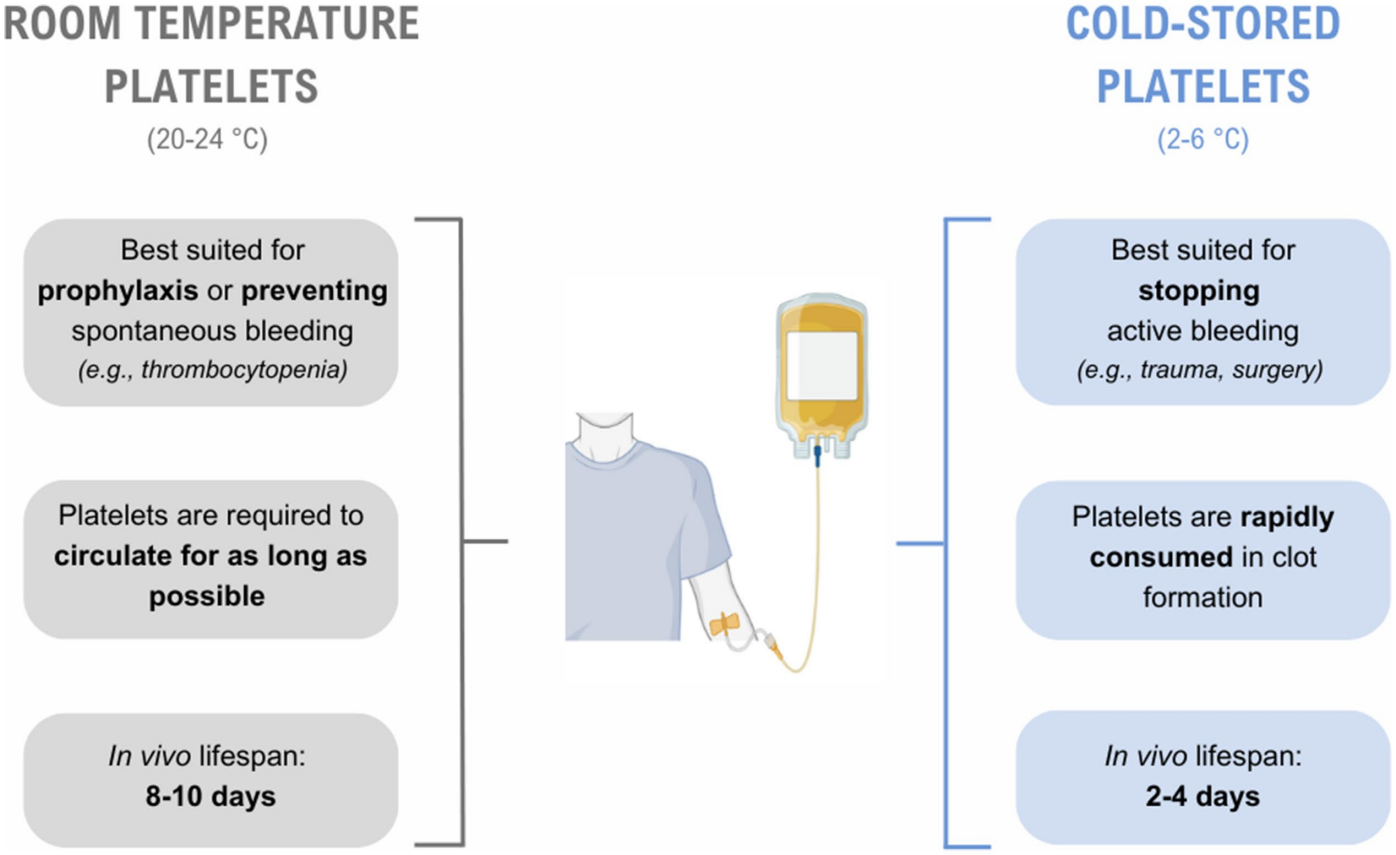
Comparison of the intended clinical use of room temperature and cold-stored platelets. Platelet components may be transfused to patients to prevent or stop bleeding. Room temperature platelets have longer *in vivo* circulation (2), and are best suited for prophylaxis. While cold-stored platelets have reduced circulation time, they are able to correct and shorten bleeding times through rapid clot formation (3), and may be the most effective treatment for patients with active bleeding. Created in BioRender. Mahajan, J (2025), https://BioRender.com/k61ekxw.

In recent years, there has been renewed interest in cold storage of platelets (2–6 °C), largely due to the potential to extend the shelf life and improve the accessibility of platelet components. This resurgence can also be attributed to the considerable strain placed on blood resources and inventories during the COVID-19 pandemic (7, 8), which have encouraged greater efforts to minimize wastage and prevent component outdating. Despite reduced *in vivo* survival, cold-stored platelets demonstrate preserved metabolic capacity, superior aggregatory properties, and maintain their clot formation and resolution functions over extended storage (9–17). Cold-stored platelets also effectively correct and shorten bleeding times (3, 4), and this rapid hemostatic effect may outweigh the need for maximal circulation in patients with critical bleeding (Figure 1).

Many of the decisions surrounding the implementation of cold-stored platelets rely on assumptions derived either from historical practices, or more recent knowledge of RT platelet storage. Specifically, significant changes in platelet collection and processing methods have occurred since the pre-1970s era of cold storage (18), meaning that historical assumptions are not reflective of current practice. Further, cold-stored platelets are now well-recognized as being phenotypically and functionally distinct from RT platelets (10, 14, 19, 20), and the use of RT data to inform decisions regarding cold storage may not be appropriate. In the last 10 years, a multitude of studies have assessed the impact of cold storage on platelet quality, and it is becoming apparent that differences in platelet collection and processing, including the collection platform, storage solution, additional component transformations, and storage duration, differentially impact *in vitro* and *in vivo* parameters (21–26). However, this variability is not sufficiently recognized and must be considered in order to guide best practice. This review highlights how variations in collection and processing of cold-stored platelets affect the quality of components, and the need to re-evaluate current assumptions to support broader integration into clinical practice.

## 2 Collection, manufacturing and storage

### 2.1 Collection platform and preparation method

Platelet components may be whole-blood derived, with pooled units prepared from the buffy coats or interim platelet units (IPUs) of multiple ABO-matched donors, or from a single donor collected via apheresis (27). While the majority of the current literature is based on data from apheresis platelets (12, 20–23, 25), there is also substantial evidence regarding the effect of cold storage on pooled platelet concentrates (9–11, 14). However, to date, only a single comparative investigation has directly compared the *in vitro* quality parameters of cold-stored apheresis versus pooled platelet concentrates (28). The findings show that cold-induced changes occur to a similar extent over storage, with the exception of a higher proportion of platelets positive for the activation markers P-selectin and phosphatidylserine in pooled platelets after 21 days of storage (28). While these results are promising, and suggest that both apheresis and pooled platelets can be refrigerated without significant alterations in quality, further studies are required to support and expand upon this evidence.

Apheresis collection platforms differentially expose platelets to varying levels of mechanical and shear stress, due to distinct cell separation methods and flow rates (29). It has been well established that these factors influence the hemostatic properties of RT-stored platelets (29, 30). Similar variability has been observed in cold-stored platelets, with significant differences in quality, metabolic activity and functional outcomes between certain collection platforms (13, 31–33). Specifically, Amicus platelet collections in both plasma and PAS show higher glucose consumption and increased lactate production, as well as reduced clot strength by day 21, exhibiting poorer performance indicators compared to platelets collected using the Trima platform (33). These findings underscore the importance of considering contextual differences in collection platforms when interpreting data. While there is a lack of *in vivo* data to suggest that these differences are clinically significant, the forthcoming results of the Chilled Platelet Study (CHIPS; NCT04834414; phase III, multicenter, international clinical trial) (34), involving sites across the United States (US) and Australia, may offer valuable insights.

### 2.2 Timing of refrigeration

Platelets are highly sensitive to changes in temperature, showing morphological, structural, metabolic and functional changes when exposed to temperatures below 15 °C (35). While some of these changes are induced rapidly, within 1–2 h of refrigeration, others accumulate over time (36, 37). FDA regulations previously mandated refrigeration of apheresis platelets within 2 h of collection (38), which has since been extended to 4 h (39, 40). These restrictions were primarily introduced to limit the time during which bacteria could proliferate at RT, prior to cold storage. However, adhering to such narrow time frames is practically and logistically challenging, particularly when collection sites are not co-located within processing centers. This time constraint contributes to product wastage as a result of preventable discards, if the refrigeration window is missed. Evidence now supports that an extension of this limit to 8 h post-collection may be possible without significant increases in bacterial growth (38), although a reduction in agonist-induced platelet aggregation is observed, compared to a 2-h hold (38, 41). Importantly, this functional decline does not seem to be clinically significant, as preliminary data shows increased aggregation response with the transfusion of cold-stored platelets in cardiac surgery patients (42). In practice, institute-specific protocols, logistical challenges and the availability of platelet components all contribute to variability in refrigeration timings, however, the standard approach in most studies has been to refrigerate platelets within 24 h of collection (9, 14, 21–23). Taken together, existing data suggests that short-term storage (between 8 and 24 hours) at RT, prior to refrigeration, is unlikely to significantly alter platelet quality or function.

This conclusion is further supported by the approach of delaying cold storage. Specifically, this strategy involves storing platelets under standard RT conditions until they approach expiry (previously investigated on day 4, day 5 and day 7 post-collection) (11, 43, 44), after which platelets are transferred to cold storage. This process allows for the availability of RT platelets for prophylactic use, with the option to maximize platelet inventories and avoid discards by conversion to cold storage. Delayed cold-stored platelets may subsequently be used to treat patients with active bleeding. Notably, delayed cold-stored platelets maintain comparable *in vitro* quality and functional parameters across 14–21 days, compared to platelets transferred to cold storage within 24 h of collection, despite prior storage at RT for at least 4 days (11, 43). Clinically, the transfusion of delayed cold-stored platelets led to effective hemostasis and no adverse events in patients with active bleeding (8). However, in a retrospective review of the data collected during this period, transfusion of delayed cold-stored platelets was associated with a higher requirement for postoperative transfusions in cardiac surgery patients, despite comparable hemostatic functionality to transfused RT platelets (44). Of note, in both instances, platelets were pathogen-inactivated, and without standard cold-stored comparators, the discrete impact of delayed cold storage versus pathogen inactivation could not be discerned. Importantly, a clinical study comparing the transfusion of delayed cold-stored platelets to RT platelets, also in cardiac surgery patients, is currently underway (PLTS-1; NCT06147531) (45). While the results of this trial will provide additional insights into the clinical benefit of delayed cold storage, it should be emphasized that this approach does not eliminate the bacterial risks underlying the current recommendation of an 8-h refrigeration window, and will likely require the need for pathogen inactivation or bacterial contamination screening.

### 2.3 Storage solution

The composition of platelet storage solutions varies across centers, and platelet components may be suspended in 100% plasma, or in platelet additive solution (PAS) with varying proportions of plasma. As shown in Table 1, there is considerable variability in the storage media used across research applications and clinical practice. It is becoming apparent that the type of storage solution differentially affects the quality of cold-stored platelets, particularly as storage progresses (21–23), which should be considered when interpreting data.

**Table 1:** Variations in collection methods and storage solutions for cold-stored platelets.

Country	Collection method	Storage solution	Purpose
Australia (10, 21)	Apheresis Trima collection platform	40% plasma/60% PAS-E	Preclinical research and CHIPS clinical trial
	Pooled platelets (4 WB collections)	30% plasma/70% PAS-E	
UK (74, 103)	Pooled platelets (4 WB collections)	35% plasma/65% PAS-E	Preclinical research
US (20, 23, 29, 68, 69)	Apheresis Trima collection platform	100% plasma or 35% plasma/65% PAS-F	Preclinical research, CHIPS clinical trial and treatment of actively bleeding patients
	Apheresis MCS collection platform	100% plasma	
	Apheresis Amicus collection platform	100% plasma or 35% plasma/65% PAS-C	
Norway (7, 42)	Apheresis Trima collection platform	37% plasma/63% PAS-E	Preclinical research and treatment of actively bleeding patients
	Pooled platelets (5 WB collections)		

PAS, Platelet Additive Solution; WB, Whole Blood; CHIPS, Chilled Platelet Study.

PAS-E is referred to as SSP+, T-PAS+, or PAS-IIIM; PAS-F is Isoplate; PAS-C is Intersol (104, 105).

While 100% plasma has been the traditional storage solution for platelets, as reported in most historical studies, the development of additive solutions since the early 2000’s has led to the licensure of PAS in many countries (46). In the US, platelet concentrates produced by many blood manufacturers are stored in 100% plasma (15, 20, 47). Previous comparisons of RT and cold-stored platelets in plasma have reported superior viscoelastic function of cold-stored platelets *in vitro* (48, 49), however this distinction is not as prominent with the storage of platelets in PAS (10, 25). This is due to the rapid decline in quality of RT platelets stored in plasma beyond 5–7 days, in comparison to storage in PAS, where the functional capacity of RT platelets is preserved well beyond their standard shelf life (17). Other *in vitro* quality parameters, such as pH and key surface glycoproteins, including GPVI, are similarly better maintained during cold storage in PAS (22). The benefits provided by PAS become particularly relevant beyond 14 days, during which the cold-induced changes to *in vitro* quality are most pronounced. However, storage in PAS reduces the concentration of plasma-derived coagulation proteins, resulting in comparatively longer clotting times compared to cold-stored platelets stored in plasma (22). As such, it is important to note that such interpretations are dependent on the specific experimental design and comparisons being made, as the choice of storage solution differentially affects *in vitro* quality and functional outcomes.

The choice of PAS may also lead to notable differences in platelet quality during cold storage. The composition of PAS can vary, and their use is dictated based on compatibility with collection platforms, regulatory requirements, and the capacity to support platelet quality over storage. Comparative studies of commonly used formulations, including PAS-F (US) and PAS-E (Australia and Europe), show that PAS-F is associated with poorer metabolic indicators, greater acidification of the component and a higher degree of storage-related activation, particularly during extended storage from day 14–21 (21). Similarly, another PAS licensed for use in the US, PAS-C, is associated with a significant loss of platelets over storage and higher microaggregate formation compared to PAS-F, with these differences being evident as early as day 5 of storage (23). However, the use of different collection platforms (Amicus system used for platelet collections in PAS-C; Trima system used for collections in PAS-F), may exacerbate these differences. *In vivo* investigations show higher post-transfusion recovery of cold-stored platelets stored in 100% plasma or PAS-C, compared with PAS-F (50), despite similar results for most *in vitro* variables investigated. These findings highlight the importance of recognizing that *in vitro* data may not always be an accurate predictor of *in vivo* outcomes. However, as *in vivo* comparisons of plasma versus PAS were limited to 10 days of cold storage, further studies with other commonly used PAS, such as PAS-E, over the extended storage period of 14–21 days are warranted. Taken together, both the use of PAS, and its specific formulation, can significantly alter the *in vitro* and *in vivo* outcomes of cold-stored platelets, underscoring the need to critically consider storage media as a key source of variability between components.

### 2.4 Irradiation

As an additional means of ensuring product safety, blood components may be irradiated to inactivate T-lymphocytes, in order to minimize the risk of transfusion-associated graft-versus-host disease (51). The use of irradiation varies considerably between countries, with only 20% of institutes universally irradiating all platelet components, while the majority of providers either selectively irradiate based on specific clinical indications, or not at all (52). Several studies have shown that irradiation performed using gamma or X-ray sources are comparable in terms of lymphocyte inactivation and impact on *in vitro* platelet quality during RT storage (53–55). Comparatively, only a single study has investigated the effect of irradiation during extended cold storage. Johnson et al., reported no differences in the metabolic, phenotypic, or functional profile of cold-stored platelets in PAS-E, with irradiation from either gamma and X-ray sources (56). However, given the differences between PAS-E and other storage solutions, these results may not be generalizable across all operational and clinical settings. Further, variability in the timing of irradiation between institutes, and prolonged periods of storage outside refrigerated conditions for component processing, should be considered as they may alter platelet quality parameters.

### 2.5 Pathogen inactivation

Cold storage minimizes the risk of bacterial proliferation in platelet components compared to RT storage (57). However, the treatment of platelets with pathogen inactivation (PI) systems offers an additional safeguard against undetected and emerging pathogens, further mitigating the risk of transfusion transmitted infections (58). Thus, PI treatment is routinely used for RT platelets in many blood centers (59, 60).

Despite the safety benefits, PI treatment contributes to storage lesions and these effects appear to be exacerbated under cold storage conditions, regardless of the system used (24–26). Currently, three PI systems have been developed for the treatment of platelet components (INTERCEPT, THERAFLEX and Mirasol), using UV light alone, or in combination with a photosensitizing agent. Studies using the INTERCEPT and THERAFLEX systems reported greater storage-related activation, as evidenced by increased phosphatidylserine exposure, higher GPIIb/IIIa activation and greater release of platelet-derived microparticles, compared to untreated cold-stored platelets (24–26). Additionally, INTERCEPT-treated platelets also exhibit significant functional differences, including higher thrombin generation, lower aggregation response and reduced clot retraction function (25, 26). Mirasol technology is routinely used in Europe and is similarly known to exacerbate platelet activation, metabolic changes and oxidative damage during short-term storage at room temperature (61, 62), however, no studies have investigated Mirasol-treated platelets during cold storage. As such, the potential implications of this approach warrant further research. While PI-treated delayed cold-stored platelets have been transfused (44), further studies are required to understand the clinical significance of this component modification under standard cold storage conditions. Taken together, the evidence to date demonstrates that PI treatment negatively affects aspects of *in vitro* platelet function and potentiates the cold storage lesion, regardless of storage duration or storage solution.

### 2.6 Storage duration

It is well established that progressive changes in platelet quality, structure and function occur during RT storage, as a result of ongoing metabolic activity (63). Comparatively, fewer deleterious changes have been observed for cold-stored platelets over the standard platelet shelf life of 5–7 days (20), encouraging investigations into the feasibility of prolonged storage durations, including up to 10, 15, and 21 days (7, 13, 23, 64). Changes in hemostatic properties accumulate as cold storage is extended, and a number of studies have demonstrated gradual metabolic changes, loss of surface glycoproteins and increase in activation markers, as well as reduced functional capacity, become increasingly evident over storage (10–13, 22). These progressive *in vitro* changes appear to be consistent with *in vivo* observations, whereby platelet recovery and survival decline as a function of cold storage duration (15).

Cold-stored platelets have received regulatory approval in the US and Norway with a shelf life of 14 days, for the treatment of active bleeding in instances when RT platelets are unavailable (7, 40). However, several *in vitro* studies have demonstrated that many platelet quality parameters are maintained throughout 21 days (10–12, 14, 22), informing the exploration of an extended storage duration during the recently completed CHIPS trial (34). Although a consensus on the maximum allowable shelf life is yet to be reached, forthcoming results of the trial are expected to inform a recommendation for an optimal storage duration that balances extended shelf life with the preservation of hemostatic function *in vivo*.

## 3 Transfusion practices

Transfusion practices are invariably different between institutions, as well as between certain patient cohorts (65), and have the potential to alter platelet function. Specifically, in trauma and critical care settings, patients are often at increased risk of hypothermia, which may impair platelet function and increase the need for additional blood components (66). As such, blood components are often infused through rapid blood warmers to minimize further reductions in body temperature. In contrast, scheduled procedures, such as cardiac surgery, may favor other temperature control strategies, such as the use of warming blankets and the manipulation of ambient temperature in operating theaters, to maintain normal patient body temperature and prevent the risk of adverse events (67). Given that the majority of literature investigating the clinical outcomes of cold-stored platelets has been specific to the setting of trauma and cardiac surgery (42, 68, 69), it is unclear if these strategies are currently being used, and if they result in differences to platelet quality parameters upon transfusion.

Rewarming platelets prior to transfusion may alter functional outcomes of cold-stored platelets. Historically, the transfusion of platelets through a blood warmer has not been recommended (70). However, subsequent studies have shown that infusion of RT platelets through blood warming devices does not impair function (71, 72). Recently, similar investigations regarding the use of blood warmers in the context of cold storage have demonstrated that the infusion of cold-stored platelets through a blood warmer has no effect on *in vitro* quality parameters (73, 74). However, the type of blood warmer used and its associated specifications may vary, including the set temperature range, infusion rate and the utilization of a pressure-based or rapid infusion system, which could differentially affect platelet quality. The potential clinical implications of warming platelets following cold storage warrants further investigation, particularly given that the extent to which this practice may already be in use remains unclear.

Given their inherent sensitivities to changes in temperature, prolonged periods of time out of cold storage may compromise platelet quality. Red cells for transfusion, which are routinely stored at 2–6 °C, are upheld to a strict set of guidelines which state that components must not be removed from their temperature controlled environment for longer than 30 min, and must be transfused within 4 h of removal (75). Comparable standards have not yet been established for cold-stored platelets. However, it is known that some cold-induced changes may be reversed upon rewarming, as has been previously demonstrated by temperature cycling, where platelets are exposed to controlled warming periods (76, 77). These prolonged periods of time out of refrigerated conditions, where platelets equilibrate toward RT, may significantly alter platelet function *in vivo*. In current clinical practice, it is likely that the window within which cold-stored platelets are transfused after removal from refrigerated conditions varies considerably between institutions. Further research specifically investigating the effect of temperature excursions on cold-stored platelets are required to determine the clinical significance of the resulting changes.

## 4 Discussion and other considerations

Renewed interest in cold storage over the past decade has highlighted both historical and emerging challenges that require further consideration. Currently, quality control specifications for the use of cold-stored platelets have not been established. While guidelines for RT storage, including minimum thresholds for platelet concentration and pH at expiry (1), may be provisionally adopted by some institutes, the development of more relevant specifications may be appropriate, given that cold-stored platelets are a fundamentally different product, with a significantly longer shelf life. Despite the lack of quality control markers for cold-stored platelets, a range of *in vitro* parameters are routinely used to assess platelet quality in preclinical research. However, it remains unclear which markers are the most informative and clinically relevant. Microfluidics models, which take into account the effects of shear blood flow and provide an *in vitro* environment which closely resembles normal physiological conditions (12), may offer insight into alternative markers of quality that best reflect the *in vivo* potential of cold-stored platelets.

### 4.1 Optimal storage conditions

Currently, the implementation of cold-stored platelets has relied upon storage practices established for conventional RT platelets, despite a lack of evidence supporting their suitability for cold storage. While it is known that cold-stored platelets are to be kept in a refrigerator at 2–6 °C, the specific storage parameters required to best maintain platelet quality remain largely undefined. Specifically, while RT platelets are stored with continuous agitation to support gas exchange, it was presumed that cold-stored platelets would not require agitation due to their reduced metabolic rate. Further, FDA guidelines state the requirement for agitation as “optional” (40), suggesting that variability in institutional practices may exist. Although a small number of studies have reported that agitation does not impact *in vitro* function or provide any benefit during cold storage, these investigations have been limited to platelets stored in plasma, or during short-term storage (5 days) (20, 47, 78). Given the impact that storage solution and duration have on platelet quality, it is unclear whether application of these findings to extended storage of platelets in PAS is suitable.

Perforated shelving is commonly used in platelet agitators to best support gas exchange during RT storage. Shelving characteristics have only recently been considered in the context of cold storage, and do not appear to have an effect on the *in vitro* quality parameters of cold-stored platelets; with similar phenotypic and functional profiles observed over 14 days (79). Of note, this study was limited to platelets stored in plasma using reduced-size platelet bags (10% of the standard storage bag volume), and the effects of downscaling volumes for cold-stored platelets have not been fully established.

To support adequate gas exchange and metabolic activity at RT, platelets are stored horizontally, in a single-layer storage configuration. In contrast, it is standard blood banking practice for red cells, stored at 2–6 °C, to be stacked in a vertical/upright orientation (80). Given that the metabolic capacity of platelets is reduced and/or altered during cold storage (9, 10, 81), stacking platelet units, either vertically like red cells, or horizontally, may be possible. However, there is potential that the reduced surface area available for gas exchange may generate hypoxic conditions that increase oxidative stress (82). Further studies exploring the requirement for single-layer storage, or the feasibility of stacking, would allow storage practices to be standardized across institutions and potentially enable the logistics of cold storage to be simplified.

Commercially available PAS have been specifically formulated to support the requirements of platelet metabolism during RT storage (83). As such, there remains significant opportunity to optimize PAS formulations specifically toward the requirements of cold-stored platelets. Recently, Shea et al., have shown the importance of taurine and purine metabolism during cold storage (12). The development of PAS containing compounds that support specific metabolic pathways during cold storage may be beneficial.

### 4.2 *In vivo* clearance

Since the shift away from cold-stored platelets, an extensive body of work has identified the mechanisms driving rapid clearance. Specifically, Hoffmeister and colleagues demonstrated that clustering of GPIbα on the platelet surface, and removal of sialic acid, facilitate rapid recognition and removal of cold-stored platelets from circulation by macrophages and hepatocytes (84–86). However, these studies were primarily conducted in murine models or using platelet-rich-plasma (PRP), with variable storage durations. As such, the findings may not be generalizable to platelet concentrates used in standard blood banking. A small number of studies have confirmed that cold-stored platelets manufactured using contemporary practices have a reduced circulation time *in vivo* (15, 50). However, these investigations have been limited to platelets stored in 100% plasma, or short-term storage for 10 days in PAS-C and PAS-F. Further studies are required to confirm the relevance of these findings beyond this time frame, and the potential implications of any post-collection modifications, including delayed cold storage and PI treatment, which may impact clearance mechanisms (26, 86).

Several avenues have been explored to improve platelet survival and prevent rapid *in vivo* clearance. These strategies include supplementation of the storage solution with compounds such as magnesium, trehalose, N-acetyl-L-cysteine, and RhoA. However, many of these studies investigate platelet components in small scale storage bags or PRP, which may not be directly translatable to cold-stored platelet concentrates in a blood bank context (87–90). While RhoA inhibition successfully prevented clearance of human platelets in a mouse model (90), these effects have yet to be confirmed in human clinical studies. Thus, while strategies to improve cold-induced clearance have been explored, there remains significant opportunity to revisit these approaches and conduct further targeted research as cold storage practices continue to evolve.

### 4.3 Cold-induced platelet aggregates

Cold storage potentiates spontaneous aggregate formation (clumping) in the storage bag (64, 91), resulting in product discard due to concerns regarding safety and suitability for transfusion. Extended periods of cold storage increase the likelihood of aggregate formation, which may negate the extension of shelf life if units are ultimately unsuitable for transfusion. It is becoming apparent that the issue of aggregate formation is more prevalent during storage in plasma, or certain types of PAS (21, 64, 92, 93). In plasma, aggregates have been observed in nearly 20% of cold-stored platelets as early as 3 days of storage (92). Aggregate formation is thought to occur as a result of the cold-induced activation of the GPIIb/IIIa receptor, particularly in plasma, due to the presence of high concentrations of fibrinogen (64). However, despite lower concentrations of plasma fibrinogen in PAS, aggregate formation has also been identified in platelets stored in PAS-F and PAS-E, although later during storage (21, 93). Regardless of storage solution, aggregate formation continues to be an issue, with recent studies reporting the identification of aggregates in approximately 10% of cold-stored platelets by day 14–21 of storage (93, 94). Of note, components with aggregates exhibit a more “activated” hemostatic profile and pronounced storage lesion-related changes (93, 94). Interestingly, Kelly et al., reported that an overall reduction in the rate of aggregate formation coincided with the provision of specific storage instructions to hospital sites, including recommendations to limit product handling and avoid excessive air flow near components (94). This indicates that further efforts are required to better understand the specific mechanisms and storage conditions contributing to aggregate formation, such that they can be minimized.

### 4.4 Donor-related attributes

It is known that certain donor-related attributes compromise platelet quality and function during RT storage, thus influencing quality parameters even before the manufacturing and storage process begins (95, 96). The impact of donor-related attributes has similarly been reported in cold-stored platelets, with high body mass index (≥29 kg/m^2^) demonstrating significant correlations with lower platelet concentration and decreased functional response *in vitro* (97). However, these platelets were stored in 100% plasma for 7 days, which, as has been emphasized, may not translate to extended storage periods or suspension of platelets in PAS. Donor sex has similarly been associated with altered functional response, with platelets from female donors exhibiting higher thrombin generation potential and higher aggregation responses, compared to platelets from males (98). However, it is unclear if the use of different collection platforms across this study may have influenced these results. Donor-related factors have also been implicated in the development of aggregates during cold storage (21, 79, 93), further demonstrating the effect of donor variability on product quality. Although distinct associations with specific donor attributes have not yet been established, studies designed to directly examine donor characteristics may be able to elucidate these differences and identify whether donors with certain characteristics are more suitable for cold storage.

### 4.5 Operational and logistical gaps

Cold-stored platelets are being increasingly integrated into clinical practice, however, their implementation does not eliminate the requirement for an inventory of RT platelets. As a result of their reduced *in vivo* survival, cold-stored platelets are typically reserved to treat active bleeding, meaning that RT platelets remain the best option for prophylaxis. During the COVID-19 pandemic, reduced hospital and donor center capacity led to significant blood shortages. As a result, several institutions explored the feasibility of implementing dual platelet inventories (both RT and cold), to meet the clinical demand for platelet components (7, 8). While Braathen et al., reported some challenges with the management and operation of two inventories, including the time required for component issuing and a higher than expected proportion of outdates, this was likely due to immediate implementation in the face of the pandemic, without the luxury of thorough planning. While implementation requires careful consideration of operational procedures and logistics, previous experiences suggest that an inventory of both RT and cold-stored platelet components can be managed with the appropriate procedures in place.

While the management of two platelet inventories is possible, it may introduce logistical complexity and contribute to increased wastage, greater operational costs and potential difficulty in ensuring the availability and correct usage of products (99). As such, recent discussions have focused on reconsidering the possibility that cold-stored platelets may have broader applications and could be potentially suitable for prophylaxis. Stolla et al. (100) suggest that the *in vivo* half-life of cold-stored platelets (approximately 1.3 days) (2), is comparable to a typical dose interval between prophylactic transfusions (1–2 days) (101, 102), indicating that the presumed shortcoming of cold-stored platelets *in vivo* may be less significant than previously assumed. However, no recent clinical trials have been performed to assess the suitability and efficacy of cold-stored platelets in the prophylactic setting. This highlights an avenue for future exploration, which is required to evaluate current assumptions and assess the possibility of broader clinical application.

Cold-stored platelets offer a promising strategy to maximize product availability and significantly reduce wastage. However, cold storage should not merely be considered as another component modification that is implemented in addition to current platelet processing methods. To establish best practice and obtain the highest quality transfusable product, recognition and optimization of the collection, manufacturing and storage practices of cold-stored platelets should be considered. To facilitate this, conscious efforts should be made by investigators to provide detailed methodological information to ensure findings can be accurately interpreted and evaluated across diverse research and clinical settings. Specifically, further research into the clinical significance of these factors could guide strategies that inform how cold-stored platelets may be tailored toward the requirements of individual patients or specific care settings.
